# Dietary Management and Mental Health Considerations in Adult Gastroparesis: A Cross‐Sectional Survey of Dietitians

**DOI:** 10.1111/jhn.70372

**Published:** 2026-11-06

**Authors:** Gemma P. Fagan, Danielle E. Bear, Miranda C. E. Lomer

**Affiliations:** ^1^ Guy's and St Thomas' NHS Foundation Trust, King's College London London UK; ^2^ Department of Nutritional Sciences King's College London London UK

**Keywords:** dietary management, eating disorders, gastroparesis, mental health, nutrition support

## Abstract

**Introduction:**

Gastroparesis is a complex gastric motility disorder for which diet is recommended as a primary management strategy. Gastroparesis is associated with significant physical and psychological burden, with anxiety and depression commonly reported. Furthermore, gastroparesis overlaps with eating disorders (EDs), complicating management. Clinical guidelines for gastroparesis management acknowledge this overlap and recommend screening for EDs, however little is known about current dietetic practice in managing this intersection. This study aimed to explore dietitians' experiences, practice and confidence in managing adult patients with gastroparesis in the context of EDs.

**Methods:**

A cross‐sectional online survey of dietitians with experience managing gastroparesis was conducted and distributed via professional networks and social media. The survey explored demographics, dietary strategies, perceived effectiveness, barriers to implementation and mental health support, particularly in relation to EDs.

**Results:**

A total of 87 surveys were completed. There was wide variation in dietary strategies used; small‐particle diets were the least commonly implemented, while artificial nutrition support was used more frequently and perceived as more effective. Confidence in managing gastroparesis varied, with particularly low confidence in screening for EDs, regardless of years of experience (Mann–Whitney *U* = 851.4, *p* = 0.790). Only 20% of respondents screen for EDs more than half the time, and just 8% reported input from a mental health professional in their multidisciplinary teams.

**Conclusion:**

A variety of dietary strategies are used by dietitians to manage gastroparesis, of which food‐first are perceived as less effective in improving symptoms and nutritional status. Screening for EDs is inconsistent, and access to mental health support is limited. This demonstrates a need for improved dietary management of gastroparesis, better mental health resources and standardised practices to improve patient care.

## Practitioner Points

- Dietary strategies to manage gastroparesis vary widely in practice, with inconsistently defined approaches potentially limiting their effectiveness.
- Artificial nutrition support is perceived as the most effective method for symptom control and nutritional management, but decisions to escalate care often prioritise clinical indicators over quality of life.
- Limited access to mental health services is a major barrier to screening and supporting patients vulnerable to EDs.

## 1 Introduction

Gastroparesis is a disorder of gastric dysmotility which presents as persistent or intermittent upper gastrointestinal symptoms, including nausea, vomiting, early satiety, bloating and/or epigastric discomfort or pain [1]. Its diagnosis relies on objective documentation of delayed gastric emptying on gastric scintigraphy or breath tests in the absence of mechanical obstruction on endoscopic examination [2]. The global prevalence varies widely, with 13.8 and 267.7 per 100,000 adults affected in the United Kingdom (UK) and the United States (US), respectively [3, 4].

Gastroparesis can occur as a secondary complication of various chronic conditions such as diabetes and Parkinson's disease, and is also recognised as a complication following gastrointestinal and cardiac surgery [5]. In many cases, no identifiable cause of gastroparesis is found, leading to a diagnosis of idiopathic gastroparesis [6].

European and US guidelines consider dietary management as an important component in treating gastroparesis [2, 7]. A “small particle diet” is recommended; however, this is based on a novel dietary approach evaluated in two small randomised controlled trials (RCTs), demonstrating significant symptom improvement in patients with diabetic gastroparesis [8, 9]. The “small particle diet” characterises foods based on their ability to be mashed with a fork into fine particles, excluding a variety of fruits, vegetables, seeds, grains and certain carbohydrates considered to contain poorly digestible particles, such as pasta and rice. It remains unclear whether this approach is routinely implemented by dietitians across the United Kingdom as a first‐line dietary management strategy.

In cases of severe weight loss, intractable vomiting or inadequate oral intake, artificial nutrition support in the form of enteral nutrition (EN) or parenteral nutrition (PN) is advised [2]. However, the evidence base underpinning these guidelines is limited and largely based on expert opinion, which may lead to variability in nutritional care. The extent of EN and PN use among patients with gastroparesis in the United Kingdom is currently unknown.

The relationship between gastroparesis and eating disorders (EDs) is complex and potentially bidirectional. Gastroparesis symptoms such as nausea, bloating and early satiety can lead to reduced food intake or avoidance, while restrictive eating may contribute to delayed gastric emptying. Altered gastric motility has been documented in individuals with anorexia and bulimia nervosa, where disordered eating itself disrupts gastrointestinal function [10]. Avoidant/Restrictive Food Intake Disorder (ARFID), characterised by avoidance of food due to sensory sensitivity or fear of adverse consequences rather than body image concerns, has also been identified in some patients with gastroparesis, often emerging after diagnosis [11]. These behaviours can closely mimic natural responses to severe gastrointestinal symptoms, complicating diagnosis and management.

European guidelines recommend screening for EDs in cases of unintentional weight loss; however, practical guidance for implementing screening in clinical practice is limited [2]. To our knowledge, dietitians' confidence in managing EDs outside of mental health settings in the United Kingdom remains unexplored.

Mental health disorders such as anxiety and depression are also common in individuals with gastroparesis [12]. Despite increasing demand, mental health services remain underfunded and overstretched, leading to significant gaps in access to specialised care [13]. As a result, dietitians may frequently assume responsibility for aspects of mental health support without adequate resources or clear referral pathways.

Given the complexity of managing gastroparesis and the limited, heterogeneous evidence base, this survey aimed to explore UK dietitians' experiences, clinical practice patterns and confidence levels in managing adult patients with gastroparesis. Specifically, the delivery and perceived effectiveness of dietary interventions, barriers to implementation, and how professional experience influences confidence and decision‐making around artificial nutrition support were investigated. Additionally, the survey examined dietitians' approaches and barriers to screening for EDs within this patient group.

## 2 Methods

### 2.1 Survey Design

The online survey was developed and administered using Qualtrics Research Core^TM^ (www.qualtrics.com). Survey content was informed by current literature and clinical guidelines relating to gastroparesis management, EDs and multidisciplinary care. The survey comprised of 30 questions across 10 sections, covering demographics, clinical experience, dietary methods and perceived effectiveness, patient outcomes, artificial nutrition support, evidence base and EDs/mental health. Questions formats included multiple‐choice, multiple‐response, matrix rating and ranking. Questions were presented in a fixed sequence, with adaptive questioning used to minimise respondent burden. All substantive questions were mandatory, with options such as “not applicable” or “prefer not to say” provided where appropriate.

The survey was piloted with eight dietitians from a range of adult specialties to improve clarity and relevance, which resulted in revisions to question wording and response formats. The full survey instrument is provided in Supplementary File 1.

### 2.2 Participants and Recruitment

Participants were eligible if they were UK Registered Dietitians with experience of managing at least one adult patient with gastroparesis in a clinical setting. As the size of this population is unknown, no formal sample size calculation was undertaken and a convenience sampling approach was used. Participants were recruited via professional networks; work emails, professional bodies (British Dietetic Association, British Society for Enteral and Parenteral Nutrition) who advertised the survey in their newsletter and on their research webpages, and social media (LinkedIn, Instagram, Facebook). The study was approved by King's College London ethics committee (MRA‐24/25‐46473). Participants received an information sheet on the survey landing page, and provided electronic informed consent obtained prior to accessing the survey questions. Participation was voluntary, responses were anonymous, and no incentives were offered.

### 2.3 Data Collection and Analysis

Data were collected between January and March 2025. This open survey was accessible via a link distributed through the aforementioned channels. No mechanisms (e.g. cookies or IP address checks) were used to prevent or identify multiple submissions; however, Qualtrics bot detection was used to identify potentially invalid responses. Responses were captured automatically within the platform.

A completed survey was defined as a questionnaire fully submitted via Qualtrics; incomplete submissions were excluded from the analysis. Responses identified as invalid during data cleaning (e.g., bot detection or ineligible responses) were also removed prior to analysis. No imputation of missing data were performed. Responses of “not applicable,” “never used” or “not relevant” were excluded from the relevant percentage, median and inferential analyses. For analysis, the five effectiveness response categories were assigned scores of 1 (“not effective at all”) to 5 (“extremely effective”). Where applicable, denominators were reported to reflect the number of respondents eligible for each analysis.

Completed surveys were exported to Microsoft Excel (Microsoft Corp.) for cleaning and descriptive analysis. Descriptive statistics (frequencies, percentages) were generated in Excel. Inferential analyses were conducted using IBM SPSS Statistics (Version 31) [14].

Categorical variables were summarised using frequencies and percentages. Continuous and ordinal variables were assessed for normality using the Shapiro–Wilk test within each subgroup. Continuous variables are presented as mean ± standard deviation (SD) if normally distributed, or median and interquartile range (IQR) if non‐normally distributed. Ordinal variables are presented as median (IQR).

Group comparisons were conducted using the Mann–Whitney *U*‐test for two independent groups, such as dietitians with ≤ 10 years experience versus ≥ 10 years experience, or those with low versus moderate to high multidisciplinary team (MDT) involvement. MDT involvement was defined by the number of professionals involved: participants reporting two or fewer were classified as having “low MDT involvement,” and those reporting three or more as having “moderate to high MDT involvement.” Statistical significance was set at *p* < 0.05.

The study was reported in accordance with the Checklist for Reporting Results of Internet E‐Surveys (CHERRIES); the completed checklist is provided in Supplementary File 2 [15].

## 3 Results

### 3.1 Respondent Characteristics and Experience

Of 152 respondents who consented to participate, 95 completed the survey (completion rate 62.5%). Following data cleaning, 87 responses were included in final analysis. Dietitians working across all four UK nations were represented in responses, with the majority working in England (Table 1). Patients with gastroparesis were encountered across a range of clinical settings and specialties (Table 1).

**Table 1:** Participant demographics and professional experience

**Demographic**	***N* = 87**		**%**
**Country of practice**			
England	75		86.2
Scotland, Northern Ireland, Wales	12		13.8
**Grade (agenda for change)**			
Band 5	7		8.0
Band 6	30		34.5
Band 7	39		44.8
Band 8a	11		12.6
**Years of experience**			
< 10	56		64.3
10–20	19		21.8
> 20	12		13.8
**Number of patients managed throughout career**			
1–5	26		29.9
6–10	22		25.3
11–20	14		16.1
≥ 21	25		28.7
**Clinical exposure to gastroparesis**			
**Clinical setting** a			
District General Hospital		46	52.9
Tertiary Hospital		42	48.3
Community/Primary Care		21	24.1
Other^b^		9	10.3
**Clinical specialty** a			
Critical Care		16	18.4
Diabetes		11	12.6
Gastroenterology		57	65.5
General/Acute Medicine		24	27.6
Home Enteral Feeding		12	13.8
Intestinal Failure		17	19.5
Primary Care/Community		10	11.5
Renal		11	12.6
Surgery		25	28.7
Other		19	21.8

^a^ Multiple responses allowed, percentages therefore do not sum to 100%.

^b^ Other includes Freelance, Private Practice, and Mental Health Service.

### 3.2 Clinical Practice Patterns in Gastroparesis Management

A variety of dietary methods were used to manage patients with gastroparesis. The most commonly used methods were oral nutrition support (ONS) (76 [87.4%]), EN (70 [80.5%]) and modified texture diet (59 [67.8%]). Small particle diets were least frequently used (17 [19.5%]). PN was perceived as the most effective method for improving nutritional status and symptoms, with a median rating of “very effective,” whereas low/modified fat diets were perceived as least effective for improving nutritional status. Low/modified fat and low/modified fibre diets were equally perceived as least effective for improving symptoms (Table 2). Among respondents who had experience managing a patient requiring EN and/or PN (72, [83%]), EN was most frequently reported as being provided “sometimes” (40 [55.6%]) or “very often” (22 [30.6%]) when clinically indicated, provision of PN was less common, with 26 [36.2%] of respondents reporting it was “never” or “rarely” provided when clinically indicated. The most commonly reported barrier to provision of EN was “refractory/intolerable symptoms e.g. pain, nausea, vomiting”, reported by 54 [75%] of respondents. Other commonly reported barriers are shown in Figure 1.

**Table 2:** Median perceived effectiveness of dietary interventions for improving nutritional status and symptoms.

Dietary method	Respondents rating effectiveness, *n* (%)^a^	Median effectiveness: nutritional status^b^	Median effectiveness: symptoms^b^
Low/modified fat diet	57 (65.5)	Not effective at all	Slightly effective
Low/modified fibre diet	68 (78.2)	Slightly effective	Slightly effective
Small particle diet	23 (26.4)	Slightly effective	Moderately effective
Modified texture diet	66 (75.9)	Slightly effective	Moderately effective
Liquid diet	72 (82.8)	Moderately effective	Moderately effective
Oral nutrition support	84 (96.6)	Moderately effective	Moderately effective
Enteral nutrition	72 (82.8)	Very effective	Moderately effective
Parenteral nutrition	32 (36.8)	Very effective	Very effective

^a^ Number and percentage of respondents selecting an effectiveness response rather than “Never used”; percentages are based on all respondents (*n* = 87).

^b^ For analysis, effectiveness responses were scored from 1 (“not effective at all”) to 5 (“extremely effective”). “Never used” responses were excluded from effectiveness analyses; median scores were calculated among respondents providing an effectiveness rating.

**Figure 1:**
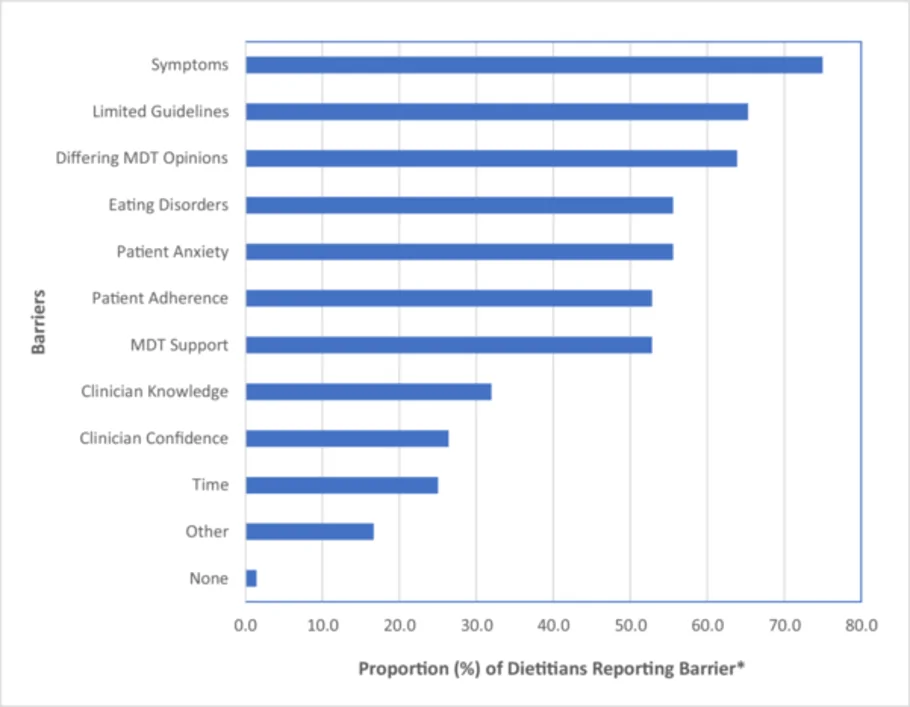
Barriers to provision of artificial nutrition support for patients with gastroparesis. *Numbers add up to more than 100% as dietitians could select more than one barrier. MDT, multi‐disciplinary team.

Most respondents (80 [91.9%]) reported that at least two different healthcare professionals were usually involved in nutritional decision‐making for patients with gastroparesis. Dietitians were most frequently reported (79 [90.8%]), whereas general practitioners, psychologists and psychiatrists were least likely to be involved (Table 3).

**Table 3:** Professionals involved in nutritional decision‐making for patients with gastroparesis.

Professional^a^	*N*	%
Dietitian	79	90.8
Specialist Nurse	13	14.9
Gastroenterologist	73	83.9
GP	1	1.1
Psychologist	2	2.3
Psychiatrist	5	5.7
Nutrition Support/Intestinal Failure Team	36	41.4
Inpatient Medical Team	50	57.5
Endocrinologist	5	5.7
Other	3	3.4

a Multiple responses allowed.

### 3.3 Challenges in Nutritional Management

Difficulties in meeting nutritional targets through dietary advice for gastroparesis were encountered frequently for energy and protein requirements, both with a median response of “very often.” Difficulty meeting fluid and micronutrient targets was less frequent with a median response of “sometimes.” When deciding to initiate artificial nutrition support, anthropometry was ranked as the most important parameter overall and quality of life as the least important. No statistically significant differences were shown between dietitians with less than 10 years' experience and those with 10 or more years' experience in how they ranked the importance of each parameter (Table 4). Common barriers to reintroducing oral intake following cessation of artificial nutrition support included patient adherence (62 [86.1%]), symptoms (59 [81.9%]), patient anxiety (56 [77.8%]) and EDs (42 [58.3%]) (Figure 2). Median confidence level in reintroducing oral intake was reported as “moderately confident.” Most participants were “not at all confident” or “slightly confident” that dietary management for gastroparesis was evidence‐based (55 [63.2%]) or supported by clinical guidelines (70 [80.4%]).

**Table 4:** Median (IQR) rankings of parameters considered when initiating artificial nutrition support, stratified by years of experience.

	<10 years’ experience Median (IQR)	>10 years’ experience Median (IQR)	*p*‐Value^a^
Anthropometry	1 (2)^b^	2 (2)	0.087
Symptom burden	3 (2)	3 (3)	0.357
Biochemistry	3 (2)	3 (2)	0.304
Hydration status	3 (3)	3.5 (2)	0.927
Quality of life	4 (2)	3.5 (3)	0.136

^a^ Mann–Whitney *U*‐test.

^b^ Lower ranks indicate higher perceived importance (1 = most important).

### 3.4 Mental Health and EDs

Among respondents for whom the question was applicable (73 [83.9%]), only 15 [20.5%] reported screening for EDs at least half the time in patients with gastroparesis who lose weight. Overall, 35 [40.2%] were not at all confident in screening for EDs in this population, with no significant association between confidence and years of professional experience (Mann–Whitney *U* = 851.4, *p* = 0.790). The most commonly reported clinician‐related barrier to ED screening was limited mental health services for onwards referral (70 [80.5%]), followed by low overall knowledge (50 [57.5%]), limited MDT support (48 [55.2%]) and limited availability of clinical guidelines and recommendations (46 [52.9%]). The most commonly reported patient‐related barrier was differing treatment priorities (e.g., symptom management versus improved nutritional status) (64 [73.6%]), followed by denial/pro‐ED beliefs (53 [60.9%]), rejection of screening (49 [56.3%]) and fear of change (49 [56.3%]). Most dietitians considered mental health support very important for patients with gastroparesis (57 [65.5%]) however its availability was reportedly low (75 [86.2%]).

**Figure 2:**
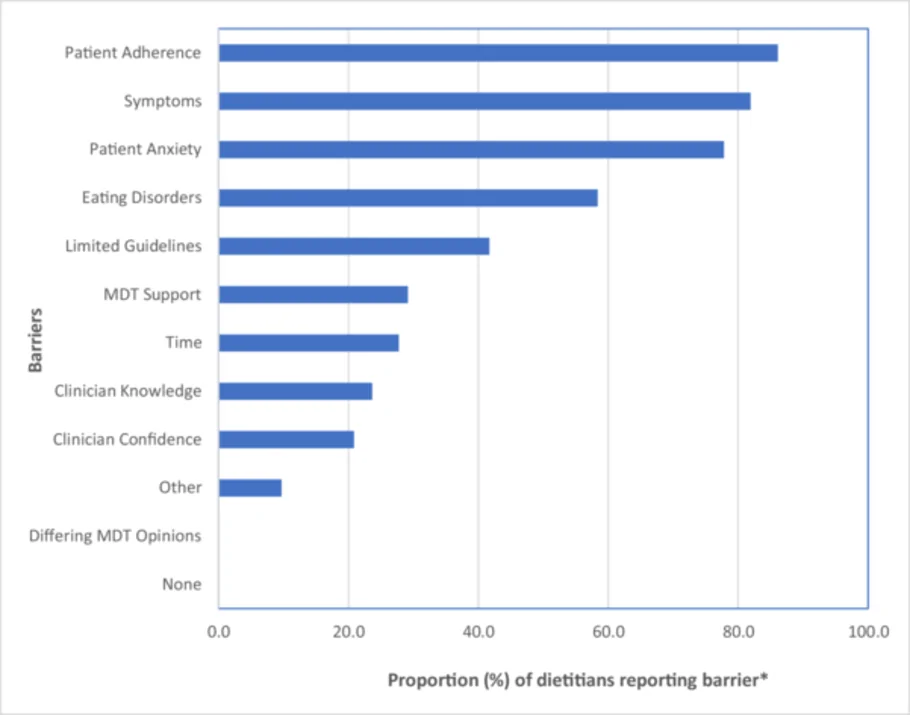
Barriers to re‐introducing oral intake in patients with gastroparesis once artificial nutrition support has ceased. *Numbers add up to more than 100% as dietitians could select more than one barrier. MDT, multi‐disciplinary team.

## 4 Discussion

To our knowledge, this cross‐sectional survey is the first to explore the experiences, clinical practice patterns and confidence levels of dietitians managing adults with gastroparesis. The findings reveal variation in dietary management strategies, low confidence in key areas and gaps between guidelines and clinical practice. Artificial nutrition support was commonly used and perceived as effective, whereas first‐line dietary approaches were underutilised or viewed as less effective. Mental health emerged as an important but under‐addressed component of care, particularly in relation to ED screening and access to psychological support.

### 4.1 Variation in Dietary Strategies and Perceived Effectiveness

Despite small‐particle diets being commonly recommended as a first‐line dietary strategy in clinical guidelines [2, 7], they were rarely implemented by dietitians. In contrast, ONS, EN and PN were more commonly employed and perceived as more effective. Modified‐texture diets were also frequently used and rated as moderately effective for symptom management, but only somewhat effective for improving nutritional status. This pattern may reflect confusion or overlap in terminology between “small‐particle” and “modified‐texture” diets. A recent scoping review highlighted a variation in definitions of dietary management strategies for gastroparesis across the existing literature, making it difficult to evaluate and compare outcomes [16]. Without standardised terminology and clearer guidance, dietitians may struggle to apply dietary recommendations with confidence. Future research should prioritise the development of consensus definitions and evaluate the clinical effectiveness of clearly defined dietary interventions.

### 4.2 Reliance on Artificial Nutrition Support

Although EN and PN were perceived as the most effective options by many dietitians, their risks must be acknowledged. Complications of EN vary by feeding route, with nasal tubes generally associated with lower risk than endoscopically paced gastrostomy or surgically placed jejunostomy tubes, which may be associated with serious complications such as buried bumper syndrome, peristomal infections, volvulus and jejonocolic fistula [17, 18]. As this survey did not capture information on specific enteral feeding routes, perceptions of effectiveness and risk could not be contextualised. Understanding which types of EN are being used is essential for future research, as effectiveness and outcomes may vary significantly by route of feeding.

Due to its associated risks, PN is only recommended where there is evidence of severe, progressive, life‐threatening malnutrition or as a bridge to appropriate therapies [19]. Furthermore, PN is known to significantly affect quality of life [20]. Despite these risks, a significant number of dietitians have experience using PN in patients with gastroparesis, and perceive it as very effective in improving nutritional status and symptoms. However, a significant proportion of respondents report that PN is never or rarely provided when clinically indicated. These findings highlight important questions: whether there is a high prevalence of severe malnutrition among gastroparesis patients in the United Kingdom, or whether a mismatch exists between guideline recommendations and dietitians' perceptions of clinical need [19].

Dietitians ranked anthropometric measures and symptom burden as the most important factors when deciding to escalate to artificial nutrition support, while quality of life was the least important. This may suggest a more medically driven decision‐making process, potentially overlooking the psychosocial impacts of interventions. To better understand how dietitians make these decisions in practice, qualitative research methods such as interviews or focus groups are needed. Such studies could explore the role of patient‐reported outcomes and identify barriers to incorporating quality of life considerations, thereby guiding more holistic and patient‐centred care.

### 4.3 Exit Strategies and Reintroduction of Oral Intake

While dietitians rated themselves as “moderately confident” in reintroducing oral intake, many also reported significant barriers which complicate this process. Although oral intake can be reintroduced safely under close dietetic supervision [21], there are currently no published protocols to guide this in practice. Furthermore, no intervention or observational studies have explored how reintroduction is managed in real‐world settings. In the absence of clear guidelines, dietitians may struggle to effectively reintroduce oral diet to a level that meets nutritional targets, particularly in patients with co‐existing psychological distress who are at risk of EDs. Further qualitative research exploring current practices and unmet needs could support the development of practical, evidence‐informed guidance for managing this transition.

### 4.4 Mental Health and EDs

Few dietitians reported involvement of a mental health professional (psychologist or psychiatrist) in nutritional decision‐making for patients with gastroparesis. Given the significant overlap between gastroparesis and disordered eating, early MDT collaboration with mental health professionals alongside dietitians may be beneficial [21]. While dietitians overwhelmingly acknowledge the importance of mental health support for this population, they consistently report limited availability of such resources.

This survey reveals that UK dietitians have low confidence levels in assessing for EDs in patients with gastroparesis. Research from Australia mirrors this, showing that dietitians working outside mental health settings often have low confidence and express a need for additional training when managing patients with EDs [22, 23].

These discrepancies in mental health support availability and low confidence may contribute to misunderstanding or dismissal of eating difficulties, potentially exacerbating patient feelings of invalidation and mistrust towards healthcare professionals [12].

### 4.5 Limitations

Several limitations of this work should be acknowledged. First, the survey did not collect detailed data on specific enteral feeding routes or protocols, thereby limiting the granularity of findings related to artificial nutrition support. Responses were also based on self‐reported practices and perceived effectiveness, which may be subject to social desirability and recall bias. As such, findings should not be interpreted as evidence of the comparative effectiveness of different approaches.

Self‐selection bias may have further influenced responses, potentially overrepresenting dietitians with a particular interest in gastroparesis. In addition, the sample size and response rate may limit the extent to which the findings reflect the wider population of UK dietitians working in this area.

The survey predominantly used closed‐ended questions, and the absence of open‐ended responses limited the ability to capture contextual detail and deeper insights into clinical decision‐making. This is particularly relevant for complex areas such as screening for EDs and the integration of mental health considerations into practice.

Finally, the survey was conducted exclusively among dietitians in the United Kingdom, potentially limiting the transferability of findings to other countries with differing healthcare infrastructures, models of mental health service provision and variations in professional scope of dietetic practice. Taken together, these limitations highlight the need for cautious interpretation of the findings which should not be considered prescriptive, particularly in relation to recommendations around clinical practice or multidisciplinary care.

## 5 Conclusion

This survey highlights considerable variation in dietary management of gastroparesis among dietitians, alongside low confidence in key areas such as ED screening and the availability of clinical guidelines. Artificial nutrition support is commonly used and perceived as effective, yet carries notable risks and uncertainties in clinical‐decision making. Mental health remains an under‐addressed but crucial aspect of care, with significant unmet needs in training and service provision. These findings underscore the urgent need for clear, evidence‐based guidelines and multidisciplinary approaches that integrate nutritional and psychological care. Further research, particularly qualitative and intervention studies, is warranted to optimise treatment strategies and improve outcomes for patients living with gastroparesis.

## Author Contributions

**Gemma P. Fagan:** conceptualization, methodology, survey design and questionnaire development, data collection, data analysis, writing – original draft, funding acquisition, final review of manuscript. **Danielle E. Bear:** conceptualization, methodology, survey design and questionnaire development, writing – review and editing, supervision, funding acquisition, final review of manuscript. **Miranda C. E. Lomer:** conceptualization, methodology, survey design and questionnaire development, writing – review and editing, supervision, funding acquisition, final review of manuscript.

## Ethics Statement

The study was approved by King's College London ethics committee (MRA‐24/25‐46473).

## Conflicts of Interest

Danielle E. Bear: Grant/Research Support from: NIHR; Speaker and consulting fees from Baxter Healthcare. Miranda C.E. Lomer: Grant/Research Support from: Crohn's and Colitis UK, Guy's and St Thomas' Charity, Guts UK, NIHR; Personal speaker fees from Janssen, Mayoly, Abbvie. The remaining author no conflicts of interest to declare.

## Supporting information


Supporting File 1

Supporting File 2

## Data Availability

The data that support the findings of this study are available on request from the corresponding author. The data are not publicly available due to privacy or ethical restrictions. The data that support the findings of this study are available from the corresponding author upon reasonable request.
