# Supplementary material for: Dietary Management and Mental Health Considerations in Adult Gastroparesis: A Cross‐Sectional Survey of Dietitians
Source: J Hum Nutr Diet. 2026 Nov 6;39(6):e70372. doi: 10.1111/jhn.70372 (PMC13742114; doi:10.1111/jhn.70372)
Supplement: Supplementary file 2 [file JHN-39-0-s002.docx]

# Supplementary File 1: Full survey instrument (Qualtrics)

Survey UK RDs and Gastroparesis

Start of Block: 1. Consent

**A survey of experience, practice and confidence of UK Dietitians managing adult patients with gastroparesis:** Thank you for taking the time to consider taking part in this research. The aim of this survey is to explore current practice, experience and confidence of UK Dietitians managing adult patients with gastroparesis across various healthcare settings. Further information on this topic will help us to identify whether current clinical guidelines are being followed, and whether there are particular barriers or enablers to providing effective dietary care to patients with gastroparesis.  Gastroparesis refers to persistent chronic nausea and vomiting with a confirmed delay in gastric emptying on gastric emptying studies. Both European and United States Gastroenterology clinical guidelines consider dietary advice as one of the primary treatment options for gastroparesis. The dietary management of this condition is complicated by the known association between eating disorders (EDs) and gastroparesis. To be eligible for participation, you must be a Dietitian currently practicing in the United Kingdom, and have experience of managing at least 1 adult patient with gastroparesis in a clinical setting. This survey is expected to take approximately 15 minutes to complete. All survey responses will be anonymous and the information obtained will remain confidential during and after the study. No personal identifiable information will be collected at any stage. You can withdraw from completing this survey at any time without giving a reason. If you require further information on this survey, please see the attached Participant Information Sheet. You can also contact [removed for anonymity] if any questions arise or if you experience any problems while completing this survey. **Please indicate that you have read and understood the information provided and that you provide your consent to your anonymous data being used for the purposes explained:**

- Yes, I consent
- No, I do not consent

End of Block: 1. Consent

Start of Block: 2. Screening

Are you a registered dietitian currently practicing in the UK?

- Yes
- No

Have you ever managed an adult patient with gastroparesis in a clinical setting?

- Yes
- No

End of Block: 2. Screening

Start of Block: 3. Setting

In which setting have you worked with patients with gastroparesis? (Please select all that apply)

- District General Hospital
- Tertiary Hospital
- Mental Health service
- Community/Primary care
- Rehabilitation service
- Freelance/Private practice
- Other (please specify) __________________________________________________

End of Block: 3. Setting

Start of Block: 4. Demographics

Which part of the UK do you work in?

- Channel Islands
- England
- Northern Ireland
- Scotland
- Wales

How many years have you been working as a Dietitian?

- 5-10 years
- 10-15 years
- 15-20 years
- >20 years

What is your Agenda for Change (AfC) grade/band? (If you work outside the NHS, please choose the most relevant option)

- Band 5
- Band 6
- Band 7
- Band 8a or above
- Other (please specify) __________________________________________________

End of Block: 4. Demographics

Start of Block: 5. Experience

How many patients with gastroparesis have you managed throughout your career?

- 1
- 2-5
- 6-10
- 11-20
- 21 – 50
- >50

What specialty/specialties did you work in when you encountered a patient/patients with gastroparesis? (Please select all that apply)

- Allergy
- Bariatrics/Weight management
- Critical Care
- Diabetes
- Elderly care
- Gastroenterology
- General/Acute Medicine
- Home Enteral Feeding
- Infectious diseases
- Intestinal Failure
- Maternity care
- Neurology
- Oncology
- Primary Care/Community
- Renal
- Surgery
- Other (Please specify) __________________________________________________

In your experience of managing patients with gastroparesis, who is involved in decision making for their nutritional care e.g. commencing or ceasing artificial nutrition support? (Please select all that apply)

- Dietitian
- Specialist Nurse
- Gastroenterologist
- GP
- Psychologist
- Psychiatrist
- Nutrition Support/Intestinal failure team
- The medical team whom the patient is admitted under if inpatient
- Other (please specify) __________________________________________________

| Page Break |  |
| --- | --- |

Which of the following methods have you used when managing patients with gastroparesis? (please select all that apply)

- Low/modified fat diet
- Low/modified fibre diet
- Small particle diet
- Modified texture diet (ie. puree diet/soft and bite-sized etc.)
- Liquid diet
- Oral nutrition support
- Enteral nutrition support
- Parenteral nutrition support
- Other (please specify) __________________________________________________

Considering the patients you have managed with gastroparesis, how effective have each of the following treatments been in **improving symptoms**?

|  | Never used | Not effective at all | Slightly effective | Moderately effective | Very effective | Extremely effective |
| --- | --- | --- | --- | --- | --- | --- |
| Low/Modified fat diet |  |  |  |  |  |  |
| Low/Modified fibre diet |  |  |  |  |  |  |
| Small particle diet |  |  |  |  |  |  |
| Modified texture diet (ie. puree/soft and bite-sized etc) |  |  |  |  |  |  |
| Liquid diet |  |  |  |  |  |  |
| Oral nutrition support |  |  |  |  |  |  |
| Enteral nutrition |  |  |  |  |  |  |
| Parenteral nutrition |  |  |  |  |  |  |

Considering the patients you have managed with gastroparesis, how effective have each of the following treatments been in **improving nutritional status**?

|  | Never used | Not effective at all | Slightly effective | Moderately effective | Very effective | Extremely effective |
| --- | --- | --- | --- | --- | --- | --- |
| Low/Modified fat diet |  |  |  |  |  |  |
| Low/Modified fibre diet |  |  |  |  |  |  |
| Small particle diet |  |  |  |  |  |  |
| Modified texture diet (ie. puree/soft and bite-sized etc) |  |  |  |  |  |  |
| Liquid diet |  |  |  |  |  |  |
| Oral nutrition support |  |  |  |  |  |  |
| Enteral nutrition |  |  |  |  |  |  |
| Parenteral nutrition |  |  |  |  |  |  |

| Page Break |  |
| --- | --- |

When managing an inpatient with gastroparesis, on average, how many hours do you spend caring for them in a typical week?

- 1 hour or less
- 1 - 3 hours
- 3-5 hours
- 5 - 10 hours
- >10 hours
- None of the above, I've never managed an inpatient with gastroparesis

Following provision of dietary advice for gastroparesis, how often do you encounter problems with meeting the patient's nutritional targets? Please answer for each individual nutritional target

|  | Never | Rarely | Sometimes | Very often | Always |
| --- | --- | --- | --- | --- | --- |
| Energy |  |  |  |  |  |
| Protein |  |  |  |  |  |
| Fluid |  |  |  |  |  |
| Micronutrients |  |  |  |  |  |

In your opinion, what is the most important outcome to decide whether a patient with gastroparesis requires artificial nutrition support (enteral or parenteral nutrition)? Please rank the below in order of importance, where 1 is the most important and 5 is the least important.

______ Anthropometry (body composition, weight changes, BMI etc)

______ Symptom burden

______ Quality of life

______ Biochemical markers (including renal profile, electrolytes, micronutrient status)

______ Hydration status/fluid balance

End of Block: 5. Experience

Start of Block: 8. Enteral & Parenteral

Have you ever managed a patient with gastroparesis requiring artificial nutrition support (enteral or parenteral nutrition)?

- Yes
- No

| Page Break |  |
| --- | --- |

In your experience, how often do patients with gastroparesis receive **enteral nutrition** when it is clinically indicated? (for example if a patient isn’t tolerating oral nutrition support)

- Never
- Rarely
- Sometimes
- Very often
- Always
- Not applicable: Enteral nutrition has never been indicated in a patient I've managed with gastroparesis

In your experience, how often do patients with gastroparesis receive **parenteral nutrition** when it is clinically indicated? (for example if a patient isn’t tolerating enteral nutrition support)

- Never
- Rarely
- Sometimes
- Very often
- Always
- Not applicable:Parenteral nutrition has never been indicated in a patient I’ve managed with gastroparesis

What barriers do you encounter when providing artificial nutrition support to patients with gastroparesis? (Please select all that apply)

- There are no barriers
- Limited availability of clinical guidelines and recommendations
- Limited time available for assessments and reviews
- Limited multi-disciplinary support
- Low confidence levels of clinician(s)
- Low knowledge levels of clinician(s)
- Differing opinions between MDT members
- Patient adherence with suggested management
- Refractory/intolerable symptoms e.g. pain, nausea, vomiting
- Patient anxiety
- Eating disorders/disordered eating
- Other (please specify) __________________________________________________

What barriers do you encounter when re-introducing oral diet on cessation of EN or PN? (Please select all that apply)

- There are no barriers
- Limited availability of clinical guidelines and recommendations
- Limited time available for assessments and reviews
- Limited multi-disciplinary support
- Low confidence levels of clinician(s)
- Low knowledge levels of clinician(s)
- Patient adherence with suggested management
- Refractory/intolerable symptoms e.g. pain, nausea, vomiting
- Patient anxiety
- Eating disorders/disordered eating
- Other (please specify) __________________________________________________

How confident are you in providing guidance on re-introducing oral diet when artificial nutrition support (EN or PN) is ceased in patients with gastroparesis?

- Not at all confident
- Slightly confident
- Moderately confident
- Very confident
- Extremely confident
- Not applicable, I've never experienced this

End of Block: 8. Enteral & Parenteral

Start of Block: 9. Evidence base and clinical guidelines

How confident are you that the dietary management you provide to patients with gastroparesis is evidence-based?

- Not at all confident
- Slightly confident
- Moderately confident
- Very confident
- Extremely confident

How confident are you that the dietary management you provide to patients with gastroparesis is supported by clinical guidelines?

- Not at all confident
- Slightly confident
- Moderately confident
- Very confident
- Extremely confident

End of Block: 9. Evidence base and clinical guidelines

Start of Block: 10. Eating Disorders, Disordered Eating & Mental Health

If a patient with gastroparesis loses weight do you complete an assessment for eating disorders/disordered eating?

- Never
- Sometimes
- About half the time
- Most of the time
- Always
- Not relevant - I work in Eating Disorders
- Not relevant - I've never experienced this

=IF(A4="Never",1,

IF(A4="Sometimes",2,

IF(A4="About half the time",3,

IF(A4="Most of the time",4,

IF(A4="Always",5,

"")))))

How confident are you in assessing for eating disorders/disordered eating in patients with gastroparesis who lose weight?

- Not at all confident
- Slightly confident
- Moderately confident
- Very confident
- Extremely confident

=IF(C4="Not at all confident",1,

IF(C4="Slightly confident",2,

IF(C4="Moderately confident",3,

IF(C4="Very confident",4,

IF(C4="Extremely confident ",5,

"")))))

In your opinion, what are the clinician-related barriers to assessing for Eating Disorders in patients with gastroparesis who experience weight loss? (Please select all that apply)

- There are no barriers
- Limited availability of clinical guidelines and recommendations
- Limited time available for assessments and reviews
- Limited multi-disciplinary support
- Limited mental health services available for onwards referral
- Low overall knowledge
- Low confidence
- Other (please specify) __________________________________________________

In your opinion, what are the patient-related barriers to assessing for Eating Disorders in patients with gastroparesis who experience weight loss?

- There are no barriers
- Rejection of screening (e.g. mistrust/fear of treatment services)
- Cultural influences (e.g. cultural norms regarding weight, food, mental illness and help-seeking, language barriers)
- Denial/Pro ED beliefs (e.g. weight stigma, ED identity, minimising/normalising symptoms)
- Fear of change (e.g. fear of losing control over eating; fear of weight gain)
- Stigma, shame or guilt (e.g. fear of discrimination; not wanting others to know; self blame)
- Differing patient priorities for treatment goals (e.g. Symptom management versus improved nutritional status)
- Other (please specify) __________________________________________________

=IF(ISNUMBER(SEARCH("Other", $O4)), 1, 0)

How important is mental health support for patients with gastroparesis?

- Not important
- Slightly important
- Fairly important
- Important
- Very important

How would you rate the current mental health support available to patients with gastroparesis?

- Very poor
- Poor
- Fair
- Good
- Very good

If you have any additional comments on this survey, please document below. This is optional and not a requirement.

________________________________________________________________

________________________________________________________________

________________________________________________________________

________________________________________________________________

________________________________________________________________

End of Block: 10. Eating Disorders, Disordered Eating & Mental Health

=IF(ISNUMBER(SEARCH("Other", $M4)), 1, 0)
